# Domestic dog demographic structure and dynamics relevant to rabies control planning in urban areas in Africa: the case of Iringa, Tanzania

**DOI:** 10.1186/1746-6148-8-236

**Published:** 2012-12-05

**Authors:** Alena S Gsell, Darryn L Knobel, Rudovick R Kazwala, Penelope Vounatsou, Jakob Zinsstag

**Affiliations:** 1 Aquatic Ecology, Netherlands Institute of Ecology (NIOO-KNAW), Wageningen, The Netherlands; 2 Department of Veterinary Tropical Diseases, University of Pretoria, Pretoria, South Africa; 3 Institute for Biodiversity Animal Health and Comparative Medicine College of Medical Veterinary and Life Sciences, University of Glasgow, Glasgow, G12, 8QQ, UK; 4 Department of Veterinary Medicine and Public Health, Sokoine University of Agriculture, Morogoro, Tanzania; 5 Department of Epidemiology and Public Health, Swiss Tropical and Public Health Institute, Basel, Switzerland

## Abstract

**Background:**

Mass vaccinations of domestic dogs have been shown to effectively control canine rabies and hence human exposure to rabies. Knowledge of dog population demography is essential for planning effective rabies vaccination programmes; however, such information is still rare for African domestic dog populations, particularly so in urban areas. This study describes the demographic structure and population dynamics of a domestic dog population in an urban sub-Saharan African setting. In July to November 2005, we conducted a full household-level census and a cross-sectional dog demography survey in four urban wards of Iringa Municipality, Tanzania. The achievable vaccination coverage was assessed by a two-stage vaccination campaign, and the proportion of feral dogs was estimated by a mark-recapture transect study.

**Results:**

The estimated size of the domestic dog population in Iringa was six times larger than official town records assumed, however, the proportion of feral dogs was estimated to account for less than 1% of the whole population. An average of 13% of all households owned dogs which equalled a dog:human ratio of 1:14, or 0.31 dogs per household or 334 dogs km^-2^. Dog female:male ratio was 1:1.4. The average age of the population was 2.2 years, 52% of all individuals were less than one year old. But mortality within the first year was high (72%). Females became fertile at the age of 10 months and reportedly remained fertile up to the age of 11 years. The average number of litters whelped per fertile female per year was 0.6 with an average of 5.5 pups born per litter. The population growth was estimated at 10% y^-1^.

**Conclusions:**

Such high birth and death rates result in a rapid replacement of anti-rabies immunised individuals with susceptible ones. This loss in herd immunity needs to be taken into account in the design of rabies control programmes. The very small proportion of truly feral dogs in the population implies that vaccination campaigns aimed at the owned dog population are sufficient to control rabies in urban Iringa, and the same may be valid in other, comparable urban settings.

## Background

Since the 1960s, the reported incidence of canine and human rabies has increased in many countries in southern and eastern Africa [1,2] even though detection rates and reporting systems have deteriorated [3] and effective human post-exposure prophylaxis and dog rabies vaccines have become available commercially [4]. Most human rabies deaths worldwide occur in Africa and Asia, with an estimated 24 500 human deaths per year in Africa, more than 100 times the number of cases officially recorded [5]. In Tanzania, canine rabies has been reported throughout the country and is considered endemic in the Iringa district [6], where 16 persons are reported to have died of rabies at the Iringa Regional Hospital between 1999-2004.

Rabies is an acute viral encephalitis transmitted by contact with saliva of an infected carrier on broken skin [5]. Throughout most of Africa and Asia, domestic dogs are the main reservoir of rabies [7,8], well able to ensure persistence of the disease [9]. Dogs are also primarily responsible for the transmission of the virus to humans [6,10-14]. Many studies on rabies dynamics and control in Africa have been focused on wildlife hosts such as bat-eared foxes [15], black-backed [16] and side-striped jackals [17], wild dogs and spotted hyenas [18]; or on domestic dog populations in rural or periurban areas such as in north-western Tanzania [11,19-22], Zambia [23,24], Zimbabwe [25,26], Kenya [27] and Tunisia [28]. However, little is known about the dynamics of canine rabies in urban settings as only few studies have been carried out in a small number of areas. These have been limited to studies on dog demography in N’Djaména, Chad [29] and Maboloko, South Africa [30], on the incidence of rabies in domestic dogs again in N’Djaména [13], and the effectiveness and costs of urban dog mass-vaccinations in Tunis [31] and N’Djaména [32,33]. Recently it was shown that combined parenteral dog mass-vaccination campaigns with human post-exposure prophylaxis (PEP) was more cost-effective than human PEP alone [34], and parenteral dog vaccination campaigns remain the most promising tool to reduce or even eliminate the incidence of canine rabies and human rabies exposures [11,20].

Canine rabies has a surprisingly low basic reproductive rate (R_0_) of 1.05 to 1.72 worldwide [20]. For rural Tanzania, the corresponding critical vaccination threshold (P_crit_) based on R_0_ of rabies is 20-40% of the targeted population [20]. Theoretical predictions of vaccination coverage necessary to prevent rabies outbreaks vary between 20-70% [20,35]. Observed levels of vaccination coverage sufficient to control rabies depend on geographic situation and targeted population. In specific circumstances, a coverage as low as 37.5% can be sufficient [36], but in other, more interconnected populations, higher levels of coverage are necessary. However, the relatively low P_crit_ reported should be treated with some caution as herd immunity declines rapidly in the interval between vaccination campaigns due to mortality of vaccinated dogs, birth or immigration of susceptible dogs and loss of individual immunity. In order to sustain the reported P_crit_ vaccination coverage in the interval between campaigns, a higher initial coverage in the order of 70% is advisable [35]. In order to estimate realistic vaccination coverage that disrupts rabies transmission in the long-term, more information on achieved coverage of vaccination campaigns, the dog subpopulations to be targeted for vaccination, patterns of contact among dogs, and between dogs and humans is necessary [37]. As recognised by WHO [38,39] dog demography studies are key to addressing many of these knowledge gaps. Even more so as rapid changes in human and dog demographics have important implications for the dynamics and control of rabies [20]. Rates of urbanization in Africa are amongst the highest in world [40], and urban communities often are characterised by highly mobile, rapidly increasing and closely-linked human and dog populations. Therefore, gaining better understanding of dog demography and ecology in these expanding urban communities remains a high priority.

Here, we report the findings of a dog-demography study carried out in four urban wards of Iringa Municipality (Tanzania), presenting results of (a) a cross-sectional household survey of 7993 households to assess the proportion of dog owning households as well as the demographics and number of owned dogs, (b) a detailed questionnaire study in a random subset of 360 households to assess dog life-histories and confinement of dogs, (c) a central-point and a house-to-house vaccination campaign to assess the achievable vaccination coverage by different intervention strategies, followed by d) a mark-recapture transect study to estimate the proportion of truly feral dogs in the population, making use of a survey on the durability of marking methods.

## Methods

### Study site

The study took place from July to November 2005 (dry season), in four urban wards of Iringa Municipality in central Tanzania (07.7° South, 35.5° East, altitude 1600m). Iringa was selected as the study location due to accounts of rabies cases in humans and dogs, and due to reports of a sizeable population of feral dogs in the town. In 2002, the Tanzanian national household level census recorded 106 668 persons living in 24 512 households in Iringa district [41]. In early 2005, the municipal veterinary office recorded a total of 1240 dogs within Iringa municipality (personal communication, Municipal Veterinary Office). Four contiguous study wards, Ilala (IL), Makorongoni (MK), Gangilonga (GL) and Kihesa (KH), were assigned by the Municipal Veterinary Officer (out of 14 urban wards in Iringa), as no large-scale rabies vaccination campaign had been conducted in these four wards within the preceding 12 months. The four wards comprised an area of 8.69km^2^ (based on minimum convex polygon area measurement in Arcview Gis 3.2) and accounted for one-third of the Iringa human population (34 162 of 106 668).

### Owned dog census and cross-sectional household survey

To assess the number of owned dogs and of dog-owning households (DHH) within the study wards, a full household-level census of dog ownership was carried out. Each household was visited by one of three teams. Each team was lead by an interviewer, accompanied by members of the local authority, and recorded the total number of pup (0-3 months), sub-adult (4-12 months) and adult (>12 months) dogs at each household. In addition to the full household-level census, 360 households were randomly selected for a more detailed questionnaire survey. DHHs were much less common that non dog-owning households (NHH). Therefore the random selection process was adapted to increase the probability of selection of DHHs in proportion to the NHH:DHH ratio, to ensure that a sufficient number of DHHs were included in the study. Equal numbers of households were fixed in both groups and randomly selected from NHHs and DHHs. This resulted in selection of 179 NHHs (2.6% of all NHH households) and 181 DHHs (10% of all DHH households). Each selected household was visited again by one of the three teams who explained the study background and obtained verbal consent to carry out the questionnaire with the head of household or, in rare cases, another adult member of the household. If no adult person was present at the selected household, the closest household of the same category (NHH or DHH) was approached. Households were defined as communities of persons sleeping in the same compound and cooking in the same kitchen.

Prior to the questionnaire survey, the questionnaire was translated into Kiswahili and back-translated as well as piloted in Mwanama, Arusha, a comparable urban setting. The questionnaire focussed on the number, demography and fecundity of the owned dogs but also included general household information. The dog demography section of the questionnaire asked about (i) the number of dogs currently owned, (ii) sex and age of all dogs, reproductive history of females including number of litters in lifetime as well as within the last 12 months and size of the latest litter, (iii) the fate of pups (*i.e.* kept, sold, given away or died) and age of pups at that event. All information was assembled into a vertical life table (Table 1) according to Pianka [42]. Based on the number of individuals *s(x)* recorded per age-class *x* several life-history parameters could be calculated. The overall survival *l(x)* from the first age-class *x1* to a given age-class *x* was calculated as

$$l\left(x\right)=\frac{s\left(x\right)}{s\left(x1\right)}$$

the age-specific survival *p(x)* denotes the probability of surviving from a given age-class *x* to the next following age-class *(x + 1)*and was calculated as

$$p\left(x\right)=\frac{s\left(x+1\right)}{s\left(x\right)}$$

**Table 1:** Overall population demography

**age class**	**n years**	**s(x)**	**% s(x)**	**l(x)**	**p(x)**	**d(x)**	**q(x)**	**e(x)**
0-1	1	189	52.06	1.00	0.28	0.00	0.72	1.76
1-2	1	52	14.33	0.28	0.96	0.72	0.04	2.76
2-3	1	50	13.77	0.26	0.52	0.74	0.48	1.83
3-4	1	26	7.16	0.14	0.50	0.86	0.50	1.60
4-5	1	13	3.58	0.07	0.20	0.93	0.80	1.20
6+	13	33	9.09	0.01	0.00	0.99	1.00	1.00

age = age in years; n years = number of years spent in the ageclass; s(x) = number of individuals sampled per ageclass; s(x)% = percentage of sample per ageclass; l(x) = cumulative survival; p(x) = age-specific survival from age x to age x + 1; d(x) = cumulative mortality; q(x) = age-specific mortality from age x to age x + 1; e(x) = age-specific life expectancy.

*d(x)* is the overall mortality from the first age-class *x1* to a given age-class *x* and was calculated as *d*(*x*) = 1 − *l*(*x*)

*q(x)* is the age-specific mortality before reaching the next following age-class and was calculated as

$$q\left(x\right)=1-p\left(x\right)$$

And finally *e(x)* is the age-specific life expectancy at a given age-class *x*, calculated as

$$e\left(x\right)=\frac{\sum \begin{array}{c} xn\\ x \end{array}l\left(y\right)}{l\left(x\right)}$$

whereby the parameter *y* is summed from age-class *x* to age-class *xn* at the end of life. As the age-class 6+ consisted of 33 individuals that were pooled over 13 years, life history calculations for *l(x), p(x)* and *e(x)* were corrected for the number of years taken into account. Dispersal effects were not taken into account which may cause an overestimation of mortality in case of emigration-, or an overestimation of survival in case of immigration of individuals.

Fecundity of females was derived from the questionnaire data and was summarised in the females life table (Table 2) where *s(x)* shows the number of females per age-class recorded in the questionnaire, *l(x)* and *d(x)* were calculated as described above; *B(x)* shows the age-class-specific average number of pups born in the last litter per female; and *b(x)* denotes the average proportion of females breeding per age-class. The average number of female pups born per age-class *m(x)* was calculated as

$$m\left(x\right)=B\left(x\right)*b\left(x\right)*0.43$$

whereby the proportion of female pups observed among all pups was incorporated into the calculation by the constant 0.43.

**Table 2:** Female demography and fecundity

**age class**	**s(x)**	**l(x)**	**d(x)**	**b(x)**	**B(x)**	**m(x)**	** parameters**	
0-1	79	1.00	0.00	0.07	7.14	0.23	α	= 0.83y
1-2	23	0.29	0.71	0.66	4.41	1.27	ω	= 11y
2-3	23	0.29	0.71	0.61	6.47	1.74		
3-4	11	0.14	0.86	0.50	5.14	1.13		
4-5	6	0.08	0.92	1.00	5.60	2.46		
6+	14	0.01	0.99	0.14	4.09	0.24		

=ageclass; s(x) = number of females in the sample per age-class; l(x) = cumulative survival; d(x) = cumulative mortality; b(x) = average proportion of breeding females per year and age-class; B(x) = average number of offspring born in the last litter per female and age-class; m(x) = number of female pups born per female and year; α = age in years at first reproduction; ω = age at last reproduction in years.

### Vaccination campaigns and marking of accessible dogs

The dog demography study was carried out as part of a wider study on dog vaccination strategies, and exploited the opportunity of a two-day central point (CP) vaccination campaign and a 14-day house-to-house (HH) follow-up vaccination campaign to mark dogs for subsequent mark-recapture transect studies. On 12^th^ and 13^th^ August 2005, a central-point vaccination campaign was carried out in three locations within the four study wards. The CP campaign was set up following local advertising by word of mouth during the household census and by posters and loudspeaker shortly before the campaign, with dog owners bringing their dogs to one of the three central point locations. In total 1619 dogs were vaccinated against rabies (1ml Rabisin ®), 1289 of the dogs (79.6% of all vaccinated) originated from the four study wards. Each vaccinated dog was marked with a coloured plastic collar around the neck. The collars served to differentiate vaccinated from unvaccinated dogs during the transect study and were fitted with reflective material for improved visibility at night.

The CP campaign was followed-up by a HH revisit to each dog-owning household in the study wards to determine the vaccination status and accessibility of owned dogs for parenteral vaccination as well as to vaccinate and mark of all previously unvaccinated dogs within two weeks. For each dog, data were collected on the age, sex, vaccination and collaring status and all dogs that had lost the vaccination collar since the CP were re-collared. During the household visits a total of 2420 dogs were recorded and 193 previously unvaccinated dogs (8% of the study population) vaccinated and collared.

### Transects

Three transect lines were selected, aiming at a minimum area coverage of 5%. Transects were run for the wards GL (6.4km, 8% coverage), KH (4.8km, 7% coverage) and MK/IL (combined, 4.7km, 18% coverage) respectively. Each transect was run on the two days following the HH revisit campaign for each of the three areas. The routes were selected to run along parallel streets (where possible) with at least 50m buffers between streets and towards the border of the adjacent wards. Implementing such 50m buffers minimised (i) the risk of counting dogs twice (between streets) and (ii) of including dogs that originated from adjacent wards. For each transect route, four consecutive runs were driven at approximately 15km/h: two night-time transects between 22h00 and 24h00 and two day-time transects between 12h00 and 14h00. For each run, a driver and a recorder sat in the front- and two observers (with flashlights for the night transects) in the back of the car. For each dog observed within 25 m on either side of the road, a GPS reading was taken and the presence of a collar and type of restriction (free-roaming or restricted) were recorded. Observation of restricted dogs was reasonably easy as household compounds had either low or no walls, so that also dogs within compounds were easily visible from the car. Additionally, no obvious differences in shyness or flight distance of owned and potentially feral dogs were observed as long as the observers stayed in the car.

### Estimation of feral dog population

The study data were fitted to a Bayesian model modified from Kayali *et al.*[32] to estimate the ratio of feral to owned dogs in each ward. The model was based on the number of marked and unmarked dogs counted during the transect study, and took into account the proportion of owned dogs that were marked and the durability of the marking (*i.e.* collars). The ratio of feral:owned domestic dogs was calculated separately for each transect area by dividing the number of marked dogs by the overall (marked and unmarked) population of dogs. While marked dogs were all vaccinated and owned, the population of unmarked dogs consisted of (i) owned, but non-vaccinated dogs, (ii) owned and vaccinated dogs that had lost the collar and (iii) truly feral dogs, *i.e.* dogs that were neither owned nor actively fed or sheltered by individuals or the community.

Bayesian inference takes into account prior information about additional model parameters. We used prior probabilities of recapture *U* of marked dogs during transects as well as the confinement probabilities *c*_*1*_ of unmarked, and *c*_*2*_ of marked dogs (Table 3). The recapture probability per ward was defined as *U* which combines the coverage, encounter and recording probabilities.Coverage is the proportion of the area covered by the transects; encounter is the probability of encountering the dog near the owner’s house; and recording is the assumed probability that an encountered dog is also recorded. Confinement *c*_*1*_ and *c*_*2*_ denote the α and β of the beta distribution of confinement for marked and unmarked dogs. Estimates for α and β were obtained using the function beta.select in the LearnBayes package in R (cran.r-project.org). The probability of collared dogs keeping their collars over time (*i.e.* durability of marks) was estimated in a separate cohort study (data not shown). The relevant time frame between vaccination and transect was three days resulting in 86% of the dogs still carrying their vaccination collar. This probability was also included in the model. All prior probabilities of encountering and confinement were collected empirically during the questionnaire survey (confinement) or the household revisits (encountering) and were therefore independent of the transect study. The R-code and the exact model are supplied in the Additional file 1 that accompanies this manuscript, the original model can be found in the annex 1 of Kayali *et al.*[32].

**Table 3:** **Prior probabilities of recapture and of confinement for GL, KH and MK/IL**^**a**^

**Probability**	**GL**	**KH**	**MK/IL**
**Recapture p**_**t**_^**(i)**^			
***U*(range)**^**b**^	0.05-0.54	0.04-0.54	0.11-0.54
**Coverage**	0.08-0.60	0.07-0.60	0.18-0.60
**Encountering**	0.70-0.90	0.70-0.90	0.70-0.90
**Recording**	0.90-0.99	0.90-0.99	0.90-0.99
**Confinement c**_**1**_^**(i)**^			
**Median**	0.33	0.33	0.33
**90% Quantile**	0.7	0.7	0.7
**Beta (α,β)**^**(i) c**^			
**α**	1.18	1.18	1.18
**β**	2.1	2.1	2.1
**Confinement c**_**2**_^**(i)**^			
**Median**	0.52	0.52	0.52
**90% Quantile**	0.7	0.7	0.7
**Beta (α,β)**^**(i) c**^			
**α**	6.25	6.25	6.25
**β**	5.8	5.8	5.8

a: GL = Gangilonga, KH = Kihesa, MK/IL = Makorongoni/Ilala.

b: Factored in three components: coverage, encountering, and recording. See additional material for an explanation of parameters.

c: α and β were estimated by the beta.select function in R (LearnBayes package) See additional material for the code and results of the elasticity analysis.

### Projection of dog population growth

The population growth was projected by means of a Leslie matrix based on female fecundity *m(x)* in the first row and survival *p(x)* in the subdiagonal of the transition matrix [43] under the assumptions that the environment remained constant, and that, given the present age distribution, the measured survival and fecundity were fixed and independent of the population size. The transition matrix was multiplied with the population vector and after 21 iterations a stable population distribution was reached (right eigenvector of the matrix) and the reproductive value of each age-class estimated (left eigenvector of the matrix) [44]. The parameters were calculated with the Excel extension Pop-Tools under the assumption that no density effects and no emigration nor immigration took place in this population. The impact of survival and fecundity in different age-classes was assessed with an elasticity analysis.

### Data entry and statistical analysis

For quality control all data of the household survey were double-entered in MS-Access (© 2007 Microsoft Corporation, One Microsoft Way, Redmond, WA 98052-6399, USA) and analysed in SASv9 (© 2007 SAS Institute Inc., SAS Campus Drive, Cary, North Carolina 27513, USA). Bootstrap analysis was done in R (R Development Core Team) for means and 95% confidence intervals (95% CI) for the proportion of DHH, number of dogs per household and dog:human ratios. The parameters of the Bayesian model, together with their credibility intervals, were approximated with Markov chain Monte Carlo simulation using WinBUGSv1.4 (© 2000 David Spiegelhalter). ArcView GIS 3.2 was used for area measurements (ESRI, 380 New York Street, Redlands, CA 92373-8100, USA). And lastly, the Leslie matrix calculations were done in the MS Excel add-in Pop-Tools (v 2.7.5 © Greg Hood, CSIRO, Canberra, Australia).

## Results

### Demography

#### Owned dog numbers

In 7993 households in the study area, a total of 2498 dogs were recorded during the census (Table 4). An average of 13.3% (95% CI: 12.56–14.06) of the households in the four wards owned at least one dog. This corresponded to 0.31 dogs per household (95% CI: 0.28-0.33) or to an average 1:14 dog:human ratio (95% CI: 1:13-1:15).

**Table 4:** Summary of ward data recorded in the study and of municipal data from the 2002 national census

	**GL**	**KH**	**IL**	**MK**	**All study area**	**Iringa**
**Nr of HH**	1630	3013	1171	2179	7993	24512
**Nr of humans**	9975	12833	3868	7486	34162	106668
**Nr of dogs**	1011	959	167	361	2498	na
**DHH in % (mean, 95% CIs)**	22.45 (20.43-24.42)	15.15 13.87-16.45	7.51 6.06-9.05	7.02 5.97-8.12	13.29 12.56-14.06	na
**Dogs/HH (mean, 95% CIs)**	0.62 (0.54-0.70)	0.30 0.27-0.34	0.14 0.11-0.18	0.16 0.12-0.19	0.31 0.28-0.33	na
**Dog:Human (mean, 95% CIs)**	1:10 (1:11-1:9)	1:14 1:16-1:13	1:23 1:31-1:18	1:21 1:26-1:18	1:14 1:15-1:13	na
**Dogs km**^**-2**^	243	298	380	415	334	na

number of households; number of inhabitants as per 2002 national census; number of dogs recorded during the study; number of dog-owning households (DHH) in mean and 95% CI; number of dogs per household counted in mean and 95%CI; dog:human ratio in mean and 95%CI; and dog densities in number of dogs recorded per km^-2^ for each ward as well as for the study area and the municipality.

An estimate of the number of owned dogs for all urban Iringa based on an extrapolation of the overall dog:human ratio predicted 7619 owned dogs within the town (95% CI: 7111-8205), whereas municipal records assumed a total of 1240 dogs in the same area. The density of dogs in the study area amounted to 334 dogs km^-2^ (95% CI: 267.45 – 400.0). The two more central wards (MK, IL) showed a lower proportion of DHH in the order of 7-7.5% but a higher density of dogs km^-2^ than the other two more suburban wards (GL, KH). Table 4 shows more detailed data on dog numbers and densities per ward.

#### Feral dog population estimates

The approximated percentage of feral dogs was below 1% for all transect areas. In a sensitivity analysis we examined the effect of the width of the 90% Quantile (0.6, 0.7 and 0.8) on the proportion of feral dogs (see supplementary material). The proportion of ownerless dogs increased with the size of the 90% Quantile but was never higher than 1%.

#### Sex ratios

In the questionnaire survey, 340 out of 389 dogs could be sexed. Six dogs were excluded from the analysis due to missing values and 43 out of 110 pups (39%) were not sexed. Of the 340 dogs, 41.5% (141/340) were female and 58.5% (199/340) were male, resulting in an overall 1:1.4 female:male ratio. Sex ratios for adults were not significantly different to those of pups (*χ*^2^ =0.046, DF = 1, p > 0.5). Data on sex ratios collected during the HH revisits was evaluated to verify the representativeness of the results of the questionnaire study. During the HH revisits, a total of 2420 dogs were recorded. Twenty dogs were excluded from the analysis because of missing values, and 130 pups (23%) and 20 adults (1%) were not sexed. Of the remaining 2250 dogs, 43.5% (977/2250) were female and 56.5% (1273/2250) were male, resulting in a 1:1.3 female: male ratio. Again, the adults only and pups only sex-ratios were not significantly different from each other (*χ*^2^ =0.151, DF = 1, p > 0.5).

The sex-ratios found in the questionnaire corresponded with those found in the HH revisits for the adult-ratios (*χ*^2^ =0.208, DF = 1, p > 0.5) as well as within pup-ratios (*χ*^2^ =0.369, DF = 1, p > 0.5), indicating that the random sampling procedure in the DHHs had generated representative data.

#### Confinement of dogs

In Iringa, leashing of dogs was not common and the method of choice for confinement of dogs was to let the dog loose within the fenced yard. Dogs running loose in yards with broken fences were considered as not-confined. Out of 394 complete records on confinement of dogs over 24 hours, 245 records were for adult dogs. Out of these, 13% (33/245) were allowed to run loose all day, whereas 63% (154/245) were confined at all times. Another 14% (34/245) were confined for two thirds of the day, the remaining 10% (24/245) were restricted for various parts of the day. Those 14% of dogs, that were allowed to roam for 8 hours were usually released around 10 pm and confined again around 6 am. Pups and juvenile dogs were generally less often confined with 20% (30/149) running loose all day and another 29% (43/149) confined for various parts of the day and 58% (86/149) were confined all day.

### Life history

#### General life history

The questionnaire data on age and sex of 363 out of 389 dogs were converted into a vertical life table. Twenty-six adult individuals with undefined exact age were excluded. The 43 unsexed pups were allocated to the both sexes proportionally to the sex ratio estimated above, in order not to under-represent the first age-class. Age-classes 6 to 18 were pooled into age-class 6+ due to low number of individuals sampled in those age-classes (Table 1).

The dog population was young, with a mean age of 2.23 years (95% CI = 2.06–2.55/Median = 2). Recorded ages ranged from 3 days to 18 years for the dogs recorded in the questionnaire survey. At the time of the study, 30.3% of the dogs were pups (0-3 months), 21.7% sub-adults (4-12 months) and 47.9% adults (>12 months). Age-specific mortality was very high in the under one year olds (72%) and the four year olds (80%). The questionnaire data on the fate of pups born within the last 12 months showed that 52.5% (107/204) died within the first three months. The life expectancy at birth was only 1.76y but rose to 2.76y after survival of the first year. Table 1 shows a life table for all dogs regardless of their sex. No differences were observed in the survival of males and females (data not shown).

Out of the 97 surviving pups, over 80% remained at the household of the mother, 11% of the pups were sold or given away within the home-ward of the owner, and another 6% of the pups were sold or given away outside of the home-ward of the owner. Out of 103 juveniles 16.5% (17/103) died before reaching maturity. Out of the remaining 86 juveniles, an average of 41% remained in the same household, about 26% were sold or given away within the same ward while 17% were sold or given away outside of the owner’s home-ward, and 9% were considered stolen.

#### Fecundity

Females started their reproductive phase early (α = 0.83y) and their reproductive lifespan reportedly lasted up to the age of 11 years (ω). Based on the number of litters per female per age class for age-classes 2 to 6, average inter-birth intervals were estimated as one litter in 1.72 years (95% CI = 1.21-2.94y). Mean litter size was 5.5 pups (95%CI = 5.0 – 5.9) No definite whelping season was observed. A summary of female survival and fecundity is given in Table 2.

#### Projection of the population growth

The Iringa dog population was growing quickly at λ = 1.10 as estimated by the dominant eigenvalue of the life table matrix. Generation time T was 2.7 years and the replacement rate per female (R_0_) was 1.45. The instantaneous rate of increase *r = lnλ* = 0.14 shows the change in population per individual per time unit. The reproductive value was highest in age-class 2 (29%) but remained at approximately 20% up to age-class 5. Table 5 shows a summary of the parameters of the projection of the population growth. An elasticity analysis of the Leslie matrix identified the under one year olds to be the life history stage with the greatest proportional effect on the change of the dominant eigenvalue λ, accounting for almost a third of the elasticity (e = 0.31) as all elasticities sum to one [44]. Survival to age-class 2 (e = 0.2) as well as the early fecundity in the age-classes 2 (e = 0.11) and 3 (e = 0.13) also strongly influenced the population growth.

**Table 5:** Estimated age structure and population parameters

**Age (y)**	**age structure**	**r(x)**	** parameters**	
1	61.9%	9.1%	λ	= 1.10
2	15.6%	28.9%	r	= 0.14
3	13.6%	21.6%	R_0_	= 1.45
4	5.7%	18.5%	T	= 2.70
5	2.7%	19.9%		
6+	0.4%	1.9%		

age structure of the estimated population after 21 iterations of the Leslie Matrix and the estimated reproductive value per female and ageclass (r(x)) in percent; λ = population growth y^-1^; r = intrinsic growth rate; R_0 =_ net reproductive rate; T = generation time in years.

### Accessibility to vaccination

During the HH revisits, 2420 dogs were revisited and their vaccination history recorded. During this second visit, 77.8% (1883/2420) of all dogs could be identified as vaccinated, either during the study’s central point (52.5%) and house-to-house vaccination campaign (5.25%) or privately and in previous governmental campaigns (19%). In 11% of cases, the pups were considered too young to be vaccinated. In another 4% of cases, the owners were not at home and the dogs were seen but could not be reached nor their vaccination status identified without doubt. For another 3% no specific information was available. Only 1.5% of the dogs could not be vaccinated because they ran away (n = 18) or were not encountered at the owners’ house (n = 16). The proportion of dogs too sick to be vaccinated was negligible.

## Discussion

Several important findings emerged from this study that have direct relevance for the design of appropriate and effective dog rabies control strategies in urban Iringa. First, the dog population size was almost six times larger than assumed by town records; second, contrary to expectation, only a very small fraction of the observed population were feral dogs; third, survival of pups and sub adults was the major factor driving population growth; and fourth, the high birth and death rates imply a high turn-over rate and rapid decline in vaccination coverage following a single vaccination campaign.

Many of the demographic and ecological characteristics of the Iringa population were consistent with those recorded in other urban African dog populations, suggesting that these results are likely to have widespread relevance for the design of dog vaccination campaigns in urban communities throughout sub-Saharan Africa. The proportion of dog owning households found in the study (14%) was consistent with a larger study carried out in coastal and inland urban areas in Tanzania finding 7.1 to 15.1% of households keeping dogs [45] and similar to a study in a suburb of Lusaka, Zambia where 11% households owned dogs [23], but differed substantially from rural African communities, where dog ownership appears to be much more common and in the order of 16.4-23.9% of households in rural Tanzania [45] or even 53-81% of households in Machakos District, Kenya [46].

The average observed dog:human ratio of 1:10 – 1:25 was also consistent with estimates from urban areas in coastal and inland Tanzania (1:14.4-1:27.2) [45], Maboloko, Bophuthatswana (1:11) [30], in the periurban Kikambuani, Kenya (1:15) [46], in suburban Zimbabwe (1:16) [47], in N’Djaména, the capital of Chad (1:21.5) [29] and in Mutendere, Zambia (1:45) [23]. These contrast with rural areas, where dog:human ratios tend to be much lower (1:6.7 in Palabana, Zambia [23] and 1:8 in Machakos District [46]). However, despite generally low dog:human ratios, dog densities in urban areas are generally much higher than in rural areas. For example, a ten times higher density was found in periurban Kenya (Kikambuani, 110 dogs km^-2^) compared to rural areas in Machakos district [46]. The densities recorded in this study (334 dogs km^-2^) are broadly consistent with these findings. In countries outside Africa, higher urban dog densities have been reported ranging from 534-936 dogs km^-2^ in Mexico [48] and up to 3000 dogs km^-2^ in Sri Lanka [24]. This suggests that people remain tolerant of dogs even with increasing numbers and that dog numbers are probably controlled by the availability of human derived food and thus human numbers rather than by the availability of other resources.

The overall Iringa dog population was much larger than veterinary officials in Iringa assumed. The official number of 1240 dogs reported in Iringa Municipality was less than the number of dogs we counted in four out of the 14 urban wards and six times less than our extrapolation of 7619 dogs for the municipality, (based on an extrapolation from the dog:human ratio). This underestimation of the actual dog population has implications for the estimate of vaccination coverage realised in governmental vaccination campaigns and calls for the introduction of improved methods of collecting census data of the dog population.

The approximated proportion of feral dogs in town accounted for less than 1% of the entire dog population, which was surprisingly low given that Iringa had been selected as a study site on accounts of a perceived large feral dog population. These results are consistent with estimates of 1.1 – 10.6% feral dogs in N’Djaména, Chad, [32] but contrast with the 61% ownerless dogs reported in Bangalore, India [49]. This stark difference is likely to arise from the cultural role and perception of dogs, and attitudes towards killing or neglecting unwanted dogs. In Africa, dogs are generally kept with a purpose such as guarding, herding or hunting and have little cultural or religious value [50]. This is in contrast to India, where dogs have a special status in society and ownerless dogs are not only tolerated but often provided with some care from the community [51]. In Iringa, the low feral dog population suggests that food resources are scarce and do not support the development of a viable feral population. A key result of this study is that, in urban Africa, rabies can be controlled by targeting vaccination campaigns at the owned dog population as the low proportion of feral dogs has a negligible effect on the vaccination coverage realised.

The dog population growth rate (10% per annum) exceeded that of the human population (1.5% per annum) [41], and was slightly higher than that recorded in Zimbabwe [26] and Kenya [46] and a lot higher than the population growth of domestic dogs reported from rural areas of Tanzania [20]. The main determinants of population growth were pup and sub-adult survival, and to a lesser degree, early fecundity, whereas a longer lifespan seemed to have less influence. The findings suggest that the dog population is able to grow rapidly despite high sub-adult mortality, as a result of large litter sizes and relatively short generation times. It should be noted that the Leslie Matrix model used in this study is limited in that it takes neither migration nor density effects into account and a cohort study would be necessary to assess immigration and emigration frequencies. Evidence from elsewhere in Africa (N’Djaména, Chad) indicates that emigration of pups may be an important factor in population dynamics (Mindekem personal communication). However, in the Iringa study area, only a small part of pups were sold or given away outside of their birth-ward.

Overall the dog population was young with 52% of the dogs younger than one year. The mean life expectancy of the dogs in the study-area was 2.76y for both sexes after survival of the first year. This corresponds closely to findings of average life expectancies of 2.8y [46] in Machakos and 1.9 y in dogs in the Serengeti [52], but is certainly much lower than in North American dogs with a life expectancy of 4.5y [53]. The 72% mortality found within the first year exceeds the mortality reported in Machakos District [27] and the reported 57% first year mortality in N’Djaména [29].

Adult females produced 0.6 litters per year (or one litter in 1.7y), with similar intervals between litters (1.6y) to those observed in dogs in communal lands and urban areas in Zimbabwe [26,47]. The average litter size was 5.5 pups, which was slightly larger than the average 4.6 and 4.8 pups per litter recorded in Zimbabwe and the average litter size of 4.7 pups in rural Tanzania [20]. Females started breeding relatively early, with the youngest breeding female 10 months old, and reproduction was recorded up to 11 years. None of the animals encountered during the study was spayed or castrated and the lack of any apparent control of reproduction resulted in high fecundity which more than compensated for the low survival of pups and juveniles and resulted in high population growth.

With a vaccination coverage of almost 78% of owned dogs, the majority of the owned dog population was relatively easily accessible for free vaccination campaigns. This corresponds to similarly high vaccination coverage reached in urban N’Djaména at central point vaccination campaigns [32].

## Conclusions

The observed high mortality and even higher fecundity indicate a high turn-over rate in the population, which in turn leads to a rapid decline in the immunisation coverage of the dog population in the interval between vaccination campaigns. In the light of the relatively low critical vaccination threshold for rabies elimination in some areas of Tanzania [20] and the cost effectiveness of dog vaccination campaigns over post-exposure treatment [34] our results call for stringent, regularly repeated mass-vaccination campaigns targeting the owned dog population. Such repeated vaccination campaigns in comparable settings as presented in our study, should have good potential to control rabies as the owned dog population is highly accessible to vaccination and as the size of the feral dog population is negligible.

## Abbreviations

IL: Ilala; MK: Makorongoni; GL: Gangilonga; KH: Kihesa; P_crit_: Critical Vaccination Threshold; DHH: Dog Owning Household; NHH: Non Dog Owning Household; CP: Central Point Vaccination Campaign; HH: House-To-House Vaccination Campaign.

## Competing interest

The authors have declared that no competing interests exist.

## Authors’ contributions

ASG conducted the fieldwork and took the lead in writing. DLK supervised and facilitated the fieldwork and corrected the manuscript. SC initiated the project, facilitated the fieldwork and corrected the manuscript. RRK facilitated the project and proofread the manuscript. PV developed the Bayesian model. JZ facilitated and supervised the project, performed statistical analyses and corrected the manuscript. All authors have read and approved of the final version of this manuscript.

## Supplementary Material

Additional file 1 Additional material. Click here for file
