# Supplementary material for: Domestic dog demographic structure and dynamics relevant to rabies control planning in urban areas in Africa: the case of Iringa, Tanzania
Source: BMC Vet Res. 2012 Dec 5;8:236. doi: 10.1186/1746-6148-8-236 (PMC3534358; doi:10.1186/1746-6148-8-236)
Supplement: Additional file 1 — Additional material. [file 1746-6148-8-236-S1.doc]

1. **Description of the model developed to estimate the ratio of ownerless dogs to owned dogs in three zones in Iringa, Tanzania**

In four wards of Iringa (Tanzania), all dogs that were accessible to vaccination either by central point or by house-to-house vaccination, were marked with a collar. The four study wards were divided into three zones, Gangilonga (zone 1), Kihesa (zone 2), and Makorongoni-Ilala combined (zone 3). In four passages along the same transect lines per zone, data were collected on the number of collared (vaccinated) and not-collared (unvaccinated) dogs encountered. From a prior household census, the total number of owned dogs per ward was known. From an additional survey on restriction practices and the durability of collars, also the probability of restriction and of loss of collars was estimated. The Bayesian statistical model has been adapted to the Iringa study site from an earlier study in N’Djaména (Chad) by Kayali et al. [35]

In the model, *X_1t_^(i)^*and *X_2t_^(i)^* are the number of owned dogs, collared and not-collared, respectively, and *Y_t_^(i)^* is the number of ownerless (and not-collared) dogs recaptured in zone *i* and on transect passage *t*. All collared dogs were owned since ownerless dogs were not brought to the vaccination points. Not-collared dogs included both categories, owned dogs that had lost the collar and also ownerless dogs as it was not possible to distinguish them. Therefore we observed only the number of not-collared dogs Z_t_^(i)^, instead of *X_2t_^(i)^* and *Y_t_^(i)^*, where Z_t_^(i)^*= X_2t_^(i)^* *+ Y_t_^(i)^* and *X_2t_^(i)^* *as well as Y_t_^(i)^* are latent data. The total number of vaccinated (collared, owned) dogs, *M_v_^(i)^*, in each zone i was known from the full household census conducted during the study.

We assume that *X_1t_^(i)^*, *X_2t_^(i)^* and Z_t_^(i)^ follow binomial distributions with binomial recapture probabilities, *p_t1_(i)*, *p_t2_(i)* and *p_t3_(i)*, respectively; that is,

*X_1t_^(i)^ ~Bn((1 – c_1_^(i)^)*M_v_^(i)^, p_t1_ ^(i)^)*;

*X_2t_^(i)^ ~Bn((1 - c_2_^(i)^)*M_u_^(i)^, p_t2_^(i)^) and*

*Z_t_^(i)^ ~Bn((1 - c_2_^(i)^)*M_u_^(i)^ + N^(i)^, p_t3_^(i)^),*

where *c_1_^(i)^* and *c_2_^(i)^* are confinement probabilities related to zone *i* for owned collared and owned not-collared dogs, respectively; *M_u_^(i)^* is the total number of unvaccinated owned dogs; and *N^(i)^* is the total number of ownerless dogs in area *i*. To reduce the number of parameters of the model, we assumed a common recapture probability, *p_t_^(i)^* for all dogs (collared owned, not-collared owned, and not-collared ownerless), that is, *p_t1_^(i)^=p_t2_^(i)^= p_t3_^(i)^ = p_t_^(i)^*.

We estimated the parameters of the model following Bayesian inference implemented by Markov chain Monte Carlo simulation. Prior information about the model parameters was obtained from the analysis of data collected during the household census and surveys on restriction practices and durability of collars. Thus an initial estimate of the total owned dog population *M^(i)^ = M_v_^(i)^ + M_u_^(i)^* in study zone *i* was taken applying the Petersen-Bailey formula for direct sampling on captured (collared) –recaptured data observed during the household survey, that is *M^(i)^ = M_v_^(i)^ *(n_i_ + 1)/m_i_ + 1* and standard error *(M^(i)^ ) = square root[M_v_^(i)^*M_v_^(i)^(n_i_ + 1)*(n_i_ – m_i_)/(m_i_ + 1)*(m_i_ + 1)*(m_i_ + 2)]*, where *n_i_* and *m_i_* are the numbers of recaptured dogs and recaptured collared (vaccinated) dogs in the household survey in zone *i*, respectively. These estimates specified the parameters of a normal prior distribution that was adopted for *M^(i)^*. For the final model data on *M^(i)^* and *M_v_^(i)^*was collected from a full census during the household visits (manuscript p. 6 line 30).

The parameter *N^(i)^* was expressed as a proportion *aa_i_* of the total owned dogs, that is *N^(i)^ = aa_i_*M^(i)^* where logit(*aa_i_*)= *a+ e_i_.*  A Uniform prior distribution was assumed for the overall proportion *a* (on the logit scale), that is  *a~* U(0.0001, 0.1) and a Normal prior was assigned to *e_i_,*  *e_i_* ~N(0,σ^2^) representing a random variation of the proportion in zone. We also assumed that τ^2^=1/σ^2^~Ga(0.1,0.1). The parameters of the above prior distributios were chosen by combining the Petersen-Bailey estimate of the owned dogs with a rough estimate of the ownerless dog population per zone obtained from the household questionnaire.

Uniform prior distributions were also adopted for the recapture probabilities *p_t_^(i)^.* The parameters of these distributions were chosen by assuming that recapture probabilities were factored in three components: the area covered by the transect line (coverage), the probability to encounter a specific dog provided the area is covered by the transect (encountering), and the probability of the observer to actually record an encountered dog (recording). For each component, uniform priors were adopted as explained below and shown in Table 3 of the main document. The lower limit for the area coverage was calculated by dividing the area covered by the transect (allowing 25 m along each side of the line to include a part of the road as well as the yard of the compound next to the road) by the total area of the zone. The upper limit for the area coverage was based on the assumption that more than 50% of the total area was covered, as the transect passed every second parallel road, most compounds are along the roads, and at intersections parallel streets could be seen. The limits of the uniform prior for the encountering component are based on our observation that many dogs gather around their compound and could therefore be seen. It is, however, a critical point in our assumption. We concluded that recording was very high by comparing the counts of dogs recorded by the three observers who drove together along each transect line.

Finally, beta distributions were adopted for the confinement probabilities *c_1_^(i)^* and *c_2_^(i)^*, *c_1_^(i)^*~Be(a_1_^(i)^,b_1_^(i)^) and *c_2_^(i)^*~Be(a_2_^(i)^,b_2_^(i)^). The proportion of dogs that, according to the household survey, spend no time outside of the compound and were in compounds with secure fences during the survey was taken as the mean of the beta distribution. The standard error of this proportion was considered equal to the standard deviation of the prior. Table 1 shows the prior distributions of confinement probabilities.

1. **Model: Estimation of the proportion of feral dogs in a population**

**With Winbugs code**

Model - Data from all zones pooled

model {

*# X_1t_^(i)^*, *X_2t_^(i)^* and Z_t_^(i)^ follow binomial distributions with binomial recapture probabilities, *p_t1_(i)*,*p_t2_(i)* and *p_t3_(i)*, # respectively; that is,

for (i in 1:zone) {

for( t in 1 : T ) {

x1[i,t] ~ dbin(p[i,t],n1[i])

z[i,t] ~ dbin(p[i,t],n2[i])

x2[i,t] ~ dbin(p[i,t],n3[i])

# p[i,t]~dunif(pmin[i],pmax[i])

p[i,t]~dbeta(arecap[i],brecap[i])

}

# lm probability of loosing the collar

n1[i]<-round((1-c1[i])*(1-lm)*Mv[i])

n2[i]<-round((1-c2[i])*(M[i]-Mv[i])+aa[i]*M[i])

n3[i]<-round((1-c2[i])*(M[i]-Mv[i]))

logit(aa[i])<-a+rand[i]

c1[i]~dbeta(a1[i],b1[i])

c2[i]~dbeta(a2[i],b2[i])

# Parameters of the Beta distribution assigned to the confinement probabilities of marked (c2) and unmarked (c1) dogs. They expressed in terms of the mean and variances

a1[i]<-m1[i]*m1[i]*((1-m1[i])/(s1[i]*s1[i]))-m1[i]

b1[i]<-a1[i]*(1-m1[i])/m1[i]

a2[i]<-m2[i]*m2[i]*((1-m2[i])/(s2[i]*s2[i]))-m2[i]

b2[i]<-a2[i]*(1-m2[i])/m2[i]

arecap[i]<-mrecap[i]*mrecap[i]*((1-mrecap[i])/(srecap[i]*srecap[i]))-mrecap[i]

brecap[i]<-arecap[i]*(1-mrecap[i])/mrecap[i]

rand[i]~dnorm(0,tau)

}

tau~dgamma(0.1,0.1)

sigma<-1/tau

a~dunif(0.0001,0.1)

}

Data

T # no of transects per zone

x1: # no of captured marked owned

x2: # no of captured unmarked owned

z : # no of captured umnarked (owned + ownereless)

aa: proportion of stray to owned dogs

pmin/pmax : prior parameters of a Uniform distribution for the recapture probabilities

Mv : total no of vaccined(marked + owned) dogs

M : total no of owned dogs (vaccinated, marked+unvaccinated)

m1/s1: mean/sd of the prior distribution of the confinement probabililty

of marked (owned) dogs

m2/s2: mean/sd of the prior distribution of the confinement probabililty

of unmarked (owned) dogs

lm: probability of loosing the collar

list(T=4,x1=structure(.Data=c(14,17,2,3,24,18,12,11,12,10,3,3),.Dim=c(3,4))

,z=structure(.Data=c(16,14,5,2,6,3,3,1,13,18,1,4),.Dim=c(3,4)),zone=3,

mrecap=c(0.8,0.8,0.8),srecap=c(0.1,0.1,0.1),

m1=c(0.325,0.325,0.325),s2=c(0.395849,0.395849,0.395849),

m2=c(0.5180723,0.5180723,0.5180723),s1=c(0.3693691,0.3693691,0.3693691)

,Mv=c(719,826,402),M=c(1011,959,528), lm=0.14)

pmin=c(0.0504,0.0441,0.1134),pmax=c(0.54,0.5346,0.534

Inits

x2: # no of captured unmarked owned

aa: proportion of ownerless/owned

list(

arat=0.001,

c1 = c(

0.7048249938577898,0.5110506501002856,0.4185334068908934),

c2 = c(

0.7164662549432108,0.3176317120761007,0.2694882891509198),

p = structure(.Data = c(

0.2,0.2,0.2,0.2,

0.3415744049443895,0.3016802151411562,0.2868451663319444,0.2127375691434546,

0.2,0.2,0.2,0.2),

.Dim = c(3,4)),

rand = c(

-0.4156218641868892,-2.613612123466846,-1.029945024347904),

tau = 0.1534377222243299,

x2 = structure(.Data = c(

10.0,3.0,7.0,8.0,28.0,

31.0,28.0,22.0,20.0,31.0,

30.0,32.0),

.Dim = c(3,4)))

Results of the sensitivity analysis

Summary of the sensitivity analysis.

We varied the confinement and recapture probabilities from 0.2 to 0.4 and to 0.8 while keeping the other fixed.

Table 1: Relationship of median proportion of ownerless dogs in relation to confinement probability for all zones

| Sensitivity analyses | Parameter | **Parameters of the prior distributions** | | | **Posterior median (and 95% BCI) for the proportion (aa) of ownerless dogs** | | |
| --- | --- | --- | --- | --- | --- | --- | --- |
|  |  | **GL** | **KH** | **MK/IL** | **GL** | **KH** | **MK/IL** |
| 1 | Mean (standard deviation) of confinement probabilities * | 0.2 (0.4) | 0.2 (0.4) | 0.2 (0.4) | 0.027 (0.002-0.048) | 0.012 (0.003-0.023) | 0.026 (0.0004-0.075) |
| 2 | Mean (standard deviation) of confinement probabilities | 0.4(0.4) | 0.4(0.4) | 0.4(0.4) | 0.001 (0.0-0.04) | 0.002 (0.0-0.02) | 0.003 (0.0- 0.07) |
| 3 | Mean (standard deviation) of confinement probabilities | 0.8(0.8) | 0.8(0.8) | 0.8(0.8) | 0.028 (0.00005-0.048) | 0.011 (0.0001-0.021) | 0.037 (0.00002-0.0078) |

a1[i]<-m1[i]*m1[i]*((1-m1[i])/(s1[i]*s1[i]))-m1[i]

b1[i]<-a1[i]*(1-m1[i])/m1[i]

Table 2 Relationship of the median proportion of ownerless dogs depending on the recapture probability.

| Sensitivity analyses | Parameter | **Parameters of the prior distributions** | | | **Posterior median (and 95% BCI) for the proportion (aa) of ownerless dogs** | | |
| --- | --- | --- | --- | --- | --- | --- | --- |
|  |  | **GL** | **KH** | **MK/IL** | **GL** | **KH** | **MK/IL** |
| 1 | Mean (standard deviation) of recapture probabilities ** | 0.2 (0.1) | 0.2 (0.1) | 0.2 (0.1) | 0.02 (0.0-0.072) | 0.013 (0.0-0.036) | 0.026 (0.0004-0.075) |
| 2 | Mean (standard deviation) of recapture probabilities ** | 0.4 (0.1) | 0.4 (0.1) | 0.4 (0.1) | 0.0 (0.0-0.029) | 0.0 (0.0-0.012 | 0.0 (0.0-0.046) |
| 3 | Mean (standard deviation) of recapture probabilities ** | 0.8 (0.1) | 0.8 (0.1) | 0.8 (0.1) | 0.0 (0.0-0.0001) | 0.0 (0.0-0.000005) | 0.0 (0.0-0.001) |

**arecap[i]<-mrecap[i]*mrecap[i]*((1-mrecap[i])/(srecap[i]*srecap[i]))-mrecap[i]

brecap[i]<-arecap[i]*(1-mrecap[i])/mrecap[i]

Interpretation of the sensitivity analysis.

The proportion of ownerless dogs is low in general (less than 2%). The proportion of ownerless dogs is sensitive to the confinement probability with a minimum around 0.4 and higher proportions for confinement probabilities of 0.2 and 0.8. The proportion of ownerless dogs decreases with increased recapture probabilities. It is zero for recapture probabilities higher than 0.4.

1. **Alternative non-Bayesian approach to estimate vaccination coverage and the proportion of unvaccinated dogs**

An alternative algebraic approach to the Bayesian method use is provided to further clarify the assessment of the average proportion of stray dogs and the vaccination coverage.

The following sub-populations are assessed during the household survey and the transect study. Unvaccinated owned dogs U and stray dogs S cannot be distinguished in the street. Vaccinated dogs are marked.

For a particular study zone:

| V = total vaccinated owned dogs | to be estimated from a Petersen-Bailey sample |
| --- | --- |
| U = total unvaccinated owned dogs | to be estimated from a Petersen-Bailey sample |
| S = total stray dogs (considered all as unvaccinated) | unknown to be estimated from transect study |
| C = V /(V + U + S) = overall vaccination coverage |  |
| v = observed vaccinated owned dogs | observed in house-to-house vaccination campaign |
| u = observed unvaccinated owned dogs | observed in house-to-house vaccination campaign |
| s = observed dogs (considered all as unvaccinated) | to be deducted from proportions of owned dogs |
| x = confinement probability for vaccinated owned dogs | Household survey (expressed as probability distribution) |
| y = confinement probability for unvaccinated owned dogs | Household survey (expressed as probability distribution) |

**Procedure:**

The household survey is conducted until a sufficient large number of vaccinated dogs is counted to fulfill the Petersen Bailey requirements. If 800 dogs are vaccinated (marked), we need to find 312 marked dogs for a minimal vaccination coverage of 50% and a standard error of 5% (Table 3.1).

**Table 3.**1: Petersen Bailey recapture sample sizes

| Population | Initially marked | Vacc coverage | Sample | Marked Sample | Estimated Population | S. E. of estimate |
| --- | --- | --- | --- | --- | --- | --- |
| N | M |  | n | m |  |  |
|  |  |  |  |  |  | 5% S. E. |
| 1600 | 800 | 50 | 624 | 312 | 1607 | 80 |
| 1333 | 800 | 60 | 477 | 286 | 1340 | 67 |
| 1143 | 800 | 70 | 364 | 255 | 1149 | 57 |
| 1000 | 800 | 80 | 269 | 216 | 1006 | 50 |
|  |  |  |  |  |  | 10% S. E. |
| 1600 | 800 | 50 | 352 | 176 | 1611 | 160 |
| 1333 | 800 | 60 | 265 | 159 | 1343 | 133 |
| 1143 | 800 | 70 | 200 | 140 | 1153 | 114 |
| 1000 | 800 | 80 | 145 | 116 | 1010 | 100 |

From recapture numbers we can derive the following statements:

c = v/o =v/(v+u) = observed vaccination coverage among the owned dogs (Household survey), or the proportion of marked animals at household level. The value c stands also for the vaccination coverage at household level

c = V / V + U (1)

using this vaccination coverage and the known number of marked animals M we may estimate V and U in the study zone on household level (provided the sampling is done in a random manner).

The transect study is done to estimate the proportion of stray dogs among the owned dogs. The transect covers 20% of the vaccination zone. The observed dogs in the street can be categorized as follows:

p = 0.2 * x * V = number of observed vaccinated (marked) dogs seen in the street as a proportion of

all vaccinated dogs (2)

q = 0.2 * (y * U) + 0.2 * S = the number of unmarked dogs seen in the street, composed of unvaccinated

owned dogs plus the stray dogs. These two categories cannot be distinguished.

An example of p and q from transects could look like this:

| Zone | Transect | p | q |
| --- | --- | --- | --- |
| 1 | 1 | 125 | 56 |
| 1 | 2 | 98 | 45 |
| 1 | 3 | 113 | 37 |
| 1 | 4 | 78 | 24 |
| 2 | 1 | 55 | 22 |
| 2 | 2 | 67 | 34 |
| 2 | 3 | 45 | 18 |

Ns = p + q = all observed dogs on the transect

Ns = 0.2 * ((x* V) + (y * U) + S))

U and S cannot be distinguished but U may be replaced by V from (1) and (2)

V= p/(0.2*x)

U = (V/c – V) = (p – cp)/(0.2*x*c)

Ns = p + (y * (p – cp)/(x*c) + 0.2*S

S = (Ns - p + (y * (p – cp)/(x*c))/ 0.2

S = q - (y * (p – cp)/(x*c))/ 0.2

C = V / V + U + S = overall vaccination coverage
